# Sampling Strategies in PRRS Elimination in Hungary: An Observational Study Involving Four Farrow-to-Finish Swine Herds

**DOI:** 10.3390/vetsci10090546

**Published:** 2023-08-30

**Authors:** Kinga Fornyos, László Búza, István Makkai, Ferenc Polyák, Imre Pogácsás, Luca Savoia, László Szegedi, Ádám Bálint, Szilvia Jakab, Krisztián Bányai, István Szabó

**Affiliations:** 1 Animal Health Testing Laboratory, Eurofins Vetcontrol Ltd., H-1211 Budapest, Hungary; kinga.fornyos@ftcee.eurofins.com; 2 Intervet Hungaria Ltd., Lechner Ödön Fasor 10/b, H-1095 Budapest, Hungary; laszlo.buza@yahoo.com (L.B.); istvan.makkai@merck.com (I.M.); 3 Tedej Agricultural Producing and Service Corporation, Fő út 9, H-4085 Hajdúnánás, Hungary; polyakferenc@tedejrt.hu; 4 Hajdúdorog Bocskai Agricultural Corporation, Nánási út 7, H-4087 Hajdúdorog, Hungary; pogacsasimre@freemail.hu; 5 Triagro Ltd., Klein Telep Triagro, H-4731 Tunyogmatolcs, Hungary; l.savoia.it@gmail.com; 6 Nagyhegyesi Agrár, Kűlső Újvárosi út 0358/13. Hrsz, H-4220 Hajdúböszörmény, Hungary; drszegedi64@gmail.com; 7 National Food Chain Safety Office Veterinary Diagnostic Directorate, H-1143 Budapest, Hungary; 8 Veterinary Medical Research Institute, Hungária krt 21, H-1143 Budapest, Hungary; jakab.szilvia@vmri.hu; 9 Department of Pharmacology and Toxicology, University of Veterinary Medicine, István u. 2, H-1078 Budapest, Hungary; 10 National PRRS Eradication Committee, H-1021 Budapest, Hungary; iszabodr@t-online.hu

**Keywords:** DIVA PCR, disease control, resident virus, PRRS-vaccinated-free status

## Abstract

**Simple Summary:**

Porcine reproductive and respiratory disease (PRRS), a viral cause of morbidity and mortality, seriously affects the productivity of the swine industry. Although the economic losses due to PRRS can be controlled by vaccination, disease eradication is challenging that requires concerted efforts. Hungary initiated a national PRRS eradication program in the mid-2010s and achieved significant countrywide success in all herd types by the early 2020s. Farrow-to-finish herds are particularly challenging from the perspective of disease control and prevention due to the simultaneous rearing of pigs of different ages. In this study, we present data about different sampling strategies of large farrow-to-finish swine farms. We demonstrate that our sampling strategy, where it was systematically applied, was successful, and these successes should guide future efforts of PRRS elimination programs in other regions.

**Abstract:**

PRRS elimination strategies often rely on depopulation-repopulation. However, this approach is accompanied by a long-term loss of production. With adequate control measures, such as well-designed immunization programs and technological changes along with prevalence-based laboratory testing, the virus-free status of the most vulnerable age groups in swine herds can be achieved. The most common reason for acquiring PRRSV at large farrow-to-finish swine farm units is that the previously settled fattening pigs serve as a source of infection for the newly reared PRRS-free animals. Following such unwanted events, PRRSV may persist in an affected establishment for several years. In this observational study, we selected four farrow-to-finish type swine herds. We implemented different laboratory testing protocols to find the most optimal solution for a successful PRRS elimination program. To aid our objectives, we used a DIVA PCR technique. The PRRS DIVA PCR assay is a fast, reliable method to identify sows shedding farm-specific PRRSV strain(s). As a result of elimination efforts at the sentinel pig herds, we found that reliable detection of wild-type PRRSV shedding among sows requires sampling at least three weaned piglets per litter. The strict adherence to this sampling protocol, the systematic use of laboratory methods that quickly detect the presence of wild virulent virus in the herd during the rearing period and the culling of DIVA PCR positive litters and their sows decreased the presence of the resident virus markedly. These procedures at Hungarian farrow-to-finish type farms successfully inhibited the wild-type PRRSV infection of different age groups. The results of this study demonstrate that applying this methodology together with strict biosecurity measures enabled us to reach PRRS-vaccinated-free status in large, farrow-to-finish herds within two years.

## 1. Introduction

Porcine reproductive and respiratory syndrome (PRRS) is a major infectious disease of swine causing the highest economic damage worldwide [1]. Infected pigs show clinical signs of reproductive failure and respiratory symptoms [2]. It is estimated that the average PRRS outbreak reduced the production by about 7.4% compared to the disease-free period, and it resulted in 1.92 fewer piglets per sow [3]. In Denmark, the disease causes losses of around $1 per pig [4]. In the US, PRRS causes a loss of $114.71 per sow and $4.67 per slaughtered pig [5]. In Scotland, PRRS costs the pig sector £80 per sow and £3.5 per slaughtered pig [6]. PRRS even causes significantly more damage to pigs in the world than African swine fever [7]. The impact of PRRS on farm profits was −19.1% on average and −41% in the worst case [8].

According to the above, if we estimate the losses due to PRRS infections in Hungary which produces 4 million slaughtered pigs or 170,000 breeding sows, it is close to 5 billion HUF (≈14 m€) per year [9].

In Hungary, based on traditions, 80–85% of the large-scale pig herds are farrow-to-finish type farms. Such farms are characterised by high stocking densities, negligence of everyday practices to break the chain of infection (lack of all-in-all-out on the building level) and unreliable working procedures (i.e., vaccinations). In addition to PRRSV, in a significant proportion of stocks, other respiratory (such as *M. hyopneumoniae*, APP, PCV2, atrophic rhinitis) and digestive (porcine dysentery, ileitis) infections are also common.

Formerly, in contrast to Aujeszky’s disease eradication, Differentiation of Infected from Vaccinated Animals (DIVA) vaccines and laboratory diagnostic methods were not commercially available against PRRS, therefore this situation required much broader veterinary expertise. In the course of practical implementation of the measures in a farm, effective cooperation has to be established with the pig farm managers and the staff working in each production phase. Eventually, Hungary commenced PRRS eradication of the pig population based on the territorial principle in 2014 [10,11]. The aim of the legislated program was set to eliminate the causative agent of the PRRS disease, including the vaccine virus strains, and reach the PRRS-free status of all pigs.

Among the PRRSV eradicating methods, the fastest and safest is the depopulation-repopulation, but in many cases, due to the long-term loss of production, it is not an option for producers [12]. By programmed immunizing of the infected breeding stock, and implementing the necessary technological changes until the weaning age is reached, virus-free rearing of piglets can often be accomplished [13,14]. In this case, the breeding herd—following vaccination at the level of the herd (full immunization twice for all breeding animals)—is preferably controlled by means of prevalence-based laboratory tests to verify the virus-free status of the weaned pigs [15]. Despite the earlier mentioned facts, the Hungarian large-scale farm units are prone to potential vertical and horizontal spread of the PRRS virus. According to our observations, the most common cause of infection is that the previously settled fattening groups are infecting the newly reared PRRS-free herds at the farrow-to-finish type farms. In this way, the causative agent could persist for several years in a given establishment.

Hence, it has become imperative to adopt a method good enough to prevent reinfection by interrupting the continuous transmission chain, as well as to maintain the PRRS-free status of stocks in all phases of production. The newly implemented multi-site system involves the isolation of weaned piglets, prefattening (nursery) and fattening pig rearing at separately located farms.

In the meantime, the World Organisation for Animal Health approved an international standard for the PRRS disease (Terrestrial Animal Health Code—2018), which describes that it is unnecessary to declare PRRS infection if it is the consequence of vaccination [16]. Based on these terms, a new innovative grade (PRRS-vaccinated-free, ‘VF’) was invented for the Hungarian farrow-to-finish type of large-scale breeding herds. The ‘VF’ grade is demanding to control the spread of resident or vaccine virus from the breeding unit to the offspring stocks, resulting in PRRS-free piglets. Achieving this requires crucial awareness in relation to the vertical spread of PRRSV from sows (both infected and vaccinated) to piglets during the period of gestation, farrowing and lactation. Subsequently, it must be guaranteed to maintain environmental conditions for the PRRS-free weaned piglets where vertical transmission from older generations can be avoided. In addition, these fattening stocks are prohibited to be immunized (with either live or inactivated vaccines) throughout their entire life (from birth to slaughter). Holtkamp declares this kind of production procedure as stable in relation to infection [17]. Nevertheless, if a resident virus strain is present with low prevalence in sows, it can be spread to the piglets and after, the infection may further accumulate in susceptible progeny of the next stages of production [18]. Henceforth, progression to a general, herd-level infection is plausible. For this reason, it is of great importance to be able to identify PCR-positive piglets even at the low occurrence of PRRSV during weaning, and culling them afterwards. At the same time, detecting and then culling infected sows that evaded control measures and vaccination is also assisted by monitoring the litters. Another substantial information required from a stable stock is the type of detected PRRSV i.e., either vaccine or resident virus. For this purpose, Fornyos et al. (2022) developed a PRRSV DIVA RT-PCR system to discriminate infected pigs from vaccinated ones [19].

Considering the above, ascertaining the proportion of piglets per litter that should be sampled from a ‘VF’ grade stock is a top priority to declare the weaned piglets PRRS-free. The specification of the American Association of Swine Veterinarians for a stable stock is the absence of PRRSV viremia by sampling 30 animals over four consecutive months [17]. The requirement for the ‘VF’ grade stocks is the negative PRRSV PCR result when sampling the produced weaned piglets in a given month based on 95% confidence and 2% prevalence data. Accordingly, in the case of 500 and 1000 animals per month, it is 129 and 138, respectively. Furthermore, sampling at least one piglet from each litter is obligatory.

In our paper, preliminary investigations were conducted to determine the principal monitoring strategy and control measures that should be performed for the declaration of stable and PRRS-free status of sows and weaned piglets, respectively. In addition, based on the results we applied the relevant methodology in practice by promoting and securing the ‘VF’ grade of a farrow-to-finish type breeding herd.

## 2. Materials and Methods

### 2.1. Farms and Intervention Plans

We share in this section the background of the elimination programs that include the general description of study sites, the history of how the involved herds became positive for PRRSV, and the strategic plan for PRRS elimination. Because all farms involved in the study were commercial herds that produced pigs for profit, controlled investigations could not be performed. The study is purely descriptive in nature.

**Figure 1:**
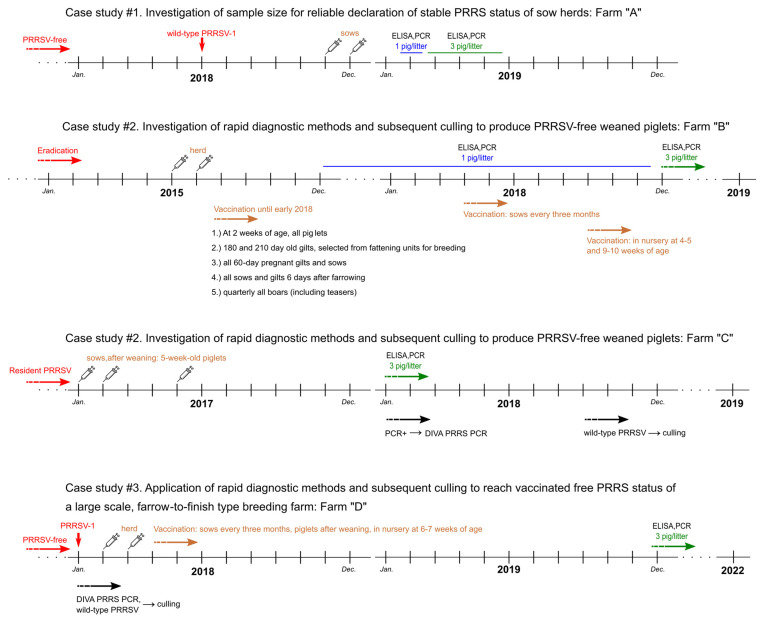
Schematic representation of the timeline and the main information including the applied vaccination and laboratory methods in the investigated pig farms (Farm “A”–“D”).

Figure 1 depicts all relevant information about the case studies, including the brief epidemiological history of the farms, the vaccination protocols, and the laboratory methods, presented in a schematic timeline.

#### 2.1.1. Case Study #1: Investigation of Sample Size for Reliable Declaration of Stable PRRS Status of Sow Herds

In order to evaluate whether taking blood samples based on 2% prevalence and 95% confidence level and a minimum of one piglet per litter, is sufficient for a reliable declaration of virus-free status, our investigations were carried out on large-scale swine Farm “A”. This farrow-to-wean type farm of 2000 sows (Danbred hybrid) provides weaned piglets for the two other stocks of the company that produce nursery pigs for the fattening farms contracted with the company (multi-site system). The farrowing was continuous on the farm while fostering was not authorised. For this particular study, the samples were taken from feeble (weaker) piglets by the attending veterinarian. The time of sampling was scheduled one week before the general age (28 days old) of weaning.

The PRRSV-free breeding unit of Farm “A” became infected with wild-type PRRSV-1 in mid-2018, confirmed by sequencing of the ORF5 gene [20]. The disease was manifested in clinical symptoms (abortions, stillbirth). All breeding animals of the 2000-sow-farm were immunized twice with a live modified attenuated vaccine (Porcilis PRRS, MSD Animal Health) on 14 November 2018 and 13 December 2018, at 4-week intervals. Later, herd closure was applied (no replacement gilts were introduced), and the offspring were not vaccinated.

Following vaccination of the entire sow population, from late January till the middle of February 2019, one individual of each litter at weaning was subjected to PRRS antibody ELISA [21,22,23] and PRRSV PCR [24] examinations from blood samples. From mid-February until late-May 2019, the process was changed from one piglet per litter to three piglets per litter.

#### 2.1.2. Case Study #2: Investigation of Rapid Diagnostic Methods and Subsequent Culling to Produce PRRSV-Free Weaned Piglets

In order to determine how to integrate the DIVA PRRS PCR assay [19] in the regular monitoring schedule, our investigations were carried out on large-scale swine Farm “B” and Farm “C”.

Farm “B” is a farrow-to-finish herd of 870 sows (Topics hybrid). In this farm, PRRSV-1 infection occurred in 2008. Between 2008 and 2015, three types of vaccines were used to reduce economic losses: two inactivated (Progressis, Ceva Santé Animale and Ingelvac PRRS KV, Boehringer Ingelheim Animal Health) and a live attenuated (Porcilis PRRS).

PRRS eradication was initiated in 2015 by mass vaccination of the herd (twice, 4 weeks apart with complete breeding (sows, gilts, boars) and offspring over 2 weeks of age). Herd vaccination was carried out for the first time between 15 and 16 June 2015 (8500 pigs were vaccinated) and for the second time between 14 and 15 July 2015 (8200 pigs were vaccinated). Porcilis PRRS was used for immunization. Subsequently, according to the eradication plan, the aim was to achieve free status through continuous production, continuous replacing of seropositive breeding sows and trying to keep the PRRSV-free status of newborn generation by the end of fattening. Their vaccination protocol was the following:

(1) At 2 weeks of age, all piglets.
(2) 180- and 210-day old gilts, selected from fattening units for breeding.
(3) all 60-day pregnant gilts and sows.
(4) all sows and gilts 6 days after farrowing.
(5) quarterly all boars (including teasers).

From the end of 2015, in order to monitor the progress of the eradication programme, one blood sample of piglet per litter 5–7 days prior to weaning was tested by ELISA and general PCR, and sentinels were introduced into the sow herd.

From September 2018, vaccination during the lactating period was stopped, and pigs in the nursery were vaccinated with Porcilis PRRS at 4–5 and 9–10 weeks of age. From April 2018, the breeding herd was immunized every three months. From 12 December 2018, the monitoring regime changed to three weaned piglets per litter 3–5 days prior to weaning.

Farm “C” is a 1400 farrow-to-slaughter breeding stock (Hypor hybrid) that was infected with PRRSV-1 in 2003. Following infection, immunization was continuously performed using live and/or inactivated PRRS vaccines according to different vaccination practices. Of the live vaccines, only the Porcilis PRRS was used. The herd was subjected to regular laboratory monitoring to detect the spread of the PRRS virus within the herd.

Initially, it appeared that the piglets could be grown up for a long period of time (7–8 months) PRRSV-free, even until the end of the fattening period. However, from the end of 2016, the resident PRRSV was found to be highly prevalent within the herd including weaned piglets, shown by ORF5 sequencing. In January, February and May 2017, all sows were re-immunized, followed by the 6–60 programme (sows were immunized at around 60th day of their pregnancy and 6th days postpartum with Porcilis PRRS). After weaning 5-week-old piglets were vaccinated with the same vaccine.

From January 2018, 5–7 days prior to each weaning, serum samples of three piglets per litter were tested PRRSV PCR. The weakest piglets (most probably infected) in the litter were chosen for blood sampling. Out of them, positive samples were tested by DIVA PRRS PCR. If the DIVA PRRS PCR indicated the presence of either wild-type or vaccine virus, sequencing was also performed. From September 2018, all sows that had litter-detected positive for the presence of wild-type PRRSV by PCR were culled together with their progenies.

Serological and virological testing of animals of different ages (preweaning, at the end of the nursery period, and at the end of the fattening period) for PRRS was performed continuously in all three farms.

#### 2.1.3. Case Study #3: Application of Rapid Diagnostic Methods and Subsequent Culling to Reach Vaccinated Free PRRS Status of a Large Scale, Farrow-to-Finish Type Breeding Farm

The following investigation was carried out in an 850-sow, farrow-to-finish type pig farm (Farm “D”, Danbred hybrid). The farm is located in a region of Hungary with the highest density of pigs which was the most affected with PRRS during 2014. At the farm, piglets intended for breeding supply were produced from the same herd. Fertilization was carried out by using purchased sperm. Farrowing was continuous and piglets stayed in the nursery for an average of 4 weeks (and no longer than 32 days). After weaning, the breeding sows were kept in the same air space but physically separated from the fattening herd. Fattening in dedicated buildings began around the age of 70 days, with a body weight of 26–30 kg, with all-in-all-out method. In each fattening building, there were 2 fattening rooms in an open space. Pigs were sold for slaughter when they reached a weight of 100 to 115 kg.

Until January 2018, the farm operated with herds free from infectious diseases that cause great economic damage (including Aujeszky’s disease, brucellosis, porcine leptospirosis, PRRS, mycoplasmosis, actinobacillosis, atrophic rhinitis, swine dysentery and scabies). In January 2018, however, the herd was infected with the PRRS virus. The management’s decision was that PRRS needs to be eliminated but not by the approach of the stock exchange. The method of elimination was the creation of a stable PRRS breeding stock with internal biosecurity measures, continuous monitoring by using specific laboratory tests and active immunization. For this purpose, the entire herd was immunized on 9–10 February 2018 and then repeated 4 weeks later. All piglets older than 1 week were also immunized.

Thereafter, all individuals of the breeding herd were immunized every 3 months, the piglets were immunized after weaning and at the age of 6–7 weeks in the nursery. In December 2020, the immunization of the breeding stock was terminated, using internal biosecurity control methods and continuous monitoring using laboratory tests, we ensured that the breeding stock remained free from the PRRS virus. Monitoring tests (ELISA and PCR) were conducted from the end of 2019 for the following groups: (i). breeding animals: semi-annual control tests; (ii). weaned piglets: 3 pigs per litter (weakest individuals in the litter) before weaning; (iii). pre-fattening piglets: 60 piglets per nursery at the end of pre-rearing (70–80 days old, still in pre-rearing resident); (iv). fattening pigs: 60 pigs at the end of the fattening phase after installation in the fattening barn.

### 2.2. Laboratory Methods and Statistical Analysis

The serological tests were carried out individually in both types of sampling method, while virological tests were performed individually in case of 1 sample/litter and the samples were pooled together regarding the 3 samples/litter. Randomly selected PCR-positive pools were analysed one by one of its components.

Laboratory methods were based on PRRS serological (ELISA [21,22]) and virological (PCR [24], and DIVA-PCR [19]) testing of animals according to the manufacturer’s instructions, and following the protocols described previously. PRRSV ORF5 was sequenced, or if it failed, the ORF7 sequence was determined, by means of a previously published protocol [20]. For phylogenetic analysis, the “similarity network” was applied [25].

Statistical analysis was carried out in R software, version 4.2.0. Fisher’s exact test was performed to compare the proportion of PCR positive results between the different methodologies applied in the investigated farms; *p*-values < 0.05 were considered statistically significant.

## 3. Results

### 3.1. Investigation of Sample Size for Reliable Declaration of Stable PRRS Status of Sow Herds

According to our results (Farm “A”), the one animal per litter sampling process at 4 weeks of age of piglets did not give any PCR positive test result in 241 litters (Table 1).

By serological tests, only 56 (7.75%) seropositive individuals were found at 4 weeks of age (Table 1). This result may indicate inadequate colostrum uptake.

From February 18 until May 21 in 2019, the number of sampled 4-week-old weaned piglets was increased to three piglets per litter. In this case, 4-week-old weaned piglets (*n* = 60), representing 20 litters out of the examined 2508 piglets, representing 836 litters (2.39%) proved to be positive by the general PRRS PCR test (Table 1). Accordingly, we found significantly more PCR-positive litters by the 3 piglet/litter sampling method (*p* < 0.01).

From the 60 PCR-positive cases, among the suckling piglets examined on March 11, 2019, in three blood samples from one litter, a PRRSV ORF7 gene was identified that was 97.3% similar to that of the farm-specific PRRS virus. This means that from the examined 836 litters farrows, only one (0.12%) litter carrying the herd-specific wild-type PRRSV was found. In all the remaining 57 PCR-positive animals, sequencing confirmed the presence of the Porcilis PRRS vaccine strain (Table 1).

### 3.2. Investigation of Rapid Diagnostic Methods and Subsequent Culling to Produce PRRSV-Free Weaned Piglets

In Farm “B” 3349 3-week-old piglets were tested in 2019. 4 litters (0.36%) proved to be PRRSV PCR positive, of which one litter was detected as farm-specific wild-type PRRSV positive, while in three litters, the Porcilis PRRS vaccine virus used for vaccination was identified (Table 2). In 2806 cases, serological tests were also carried out on serum from 3-week-old piglets, 79.86% of which were positive. This result proved much more effective in colostrum intake (management) compared to Stock “A”.

In the same year, during the nursery period, in the beginning, (about 35-day-old piglets, about 2 weeks after weaning, and around one week after vaccination) and towards the end of this period (67-day-old piglets, i.e., an additional 4 weeks in the nursery, and around second vaccination of nursery pigs) animals were also subjected to PRRS PCR and ELISA. Among the 35-day and 67-day-old piglets, 1.18% and 5.23% of piglets were positive for PRRSV (Table 2). The PCR results showed that the PRRSV detection rate significantly differed between the two sampling periods (*p* < 0.01). At the start of the nursery phase, laboratory investigations of 1438 35-day-old piglets showed that only one (0.07%) animal was infected with the farm-specific wild-type PRRSV, while in 16 samples (1.11%) Porcilis PRRS vaccine virus was detected (Table 2). Blood samples from 848 (58.97%) piglets at 35 days of age were positive for serological testing for PRRS, while 590 animals were already seronegative.

Laboratory testing at the end of the nursery period (67-day-old piglets) indicated the presence of the farm-specific wild-type PRRSV in the herd in 14 cases (0.93%). However, Porcilis PRRS was also found in 65 (4.3%) cases. The occurrence of wild-type or vaccine virus strains was not significantly different between the two age groups (*p* = 0.295).

From the 67-day-old piglets, 1423 samples were serologically tested, of which 617 (43.36%) were seropositive (Table 2). The clearance dynamics of high colostrum antibody levels are well demonstrated, although PRRSV antigens (consistent with the antigenic effect of the farm-specific wild-type PRRSV and the vaccine virus used) result in significant active antibody production due to continuous replication.

In 2018, a total of 32,115 weaned piglets were produced at the Farm “C”. Of them, 8910 (27.7%) were tested (three piglets/litter) by DIVA-PCR for the presence of PRRSV, and the positive ones were sequenced. The studied animals represented a total of 2970 farrows. Of the 8910 weaned piglets, 96 (1.08%) were PCR-positive, of which 78 (0.88%) proved to be the farm-specific wild-type virus by the DIVA PRRS PCR (Table 3). This finding was confirmed by parallel sequencing, and Porcilis PRRS was confirmed in 18 cases. In September 2018, all sows that tested positive for wild-type PRRSV in their 3-week-old piglets were culled.

In 2019, 9906 3-week-old piglets were sampled and PRRS DIVA-PCR tested. In neither of the cases was a wild-type virus detected. In 2018 and 2019, no vaccination was performed in the litters, so the vaccine virus detected in the individuals had to come from an external source (sow, infectious object, etc.) (Table 3). The maternally derived immunity level at Farm “C” showed 75.6% seropositivity, which was similar to that in Farm “B”.

Results of PRRS DIVA PCR tests in the progeny confirmed that significantly more litters were wild-virus positive in 2018 than in 2019 (*p* < 0.01) before culling positive sows plus their piglets (Table 3). Unfortunately, farm management decided to make a depopulation-repopulation process to eradicate PRRS (and other disease causing factors (mycoplasma, App, rhinitis atrophicans, swine dysentery, mange), in this way we had no chance to continue our observations. However, the above reported results confirmed that a wild-type PRRSV infection in the offspring stocks could be eliminated by this method.

### 3.3. Application of Rapid Diagnostic Methods and Subsequent Culling to Reach Vaccinated Free PRRS Status of a Large-Scale, Farrow-to-Finish Type Breeding Farm

In September 2019, at Farm “D”, we aimed to achieve the PRRS vaccinated free status (‘VF’ grade) by introducing the following measures: (i). we completed the selective culling of sows already involved in breeding at the time of PRRS virus infection in 2018; (ii). we performed systematic culling of sows giving litters infected with a resident virus based on DIVA PCR tests as well as culling of their entire litter; (iii). similarly, all pigs proved to be infected with the resident virus detected by DIVA PCR tests were culled regardless of age; (iv). the diagnostic tests were carried out in a laboratory where sample testing and data sharing were adapted to the technology; (v). the laboratory tests were regularly performed to follow-up pigs by age in the farrowing barn, the battery, and the fattener so that the sequence of the individual phases can be determined.

Table 4 summarizes the results of laboratory tests (such as ELISA, PCR, and DIVA PCR) for 3 piglets per litter starting from January 2020. At the age of the pre-weaning phase, 39% of the piglets still had maternal immunity. Only 8% of the investigated litters were PCR positive, yet, we found two litters in which the piglets were infected with the herd’s resident virus.

From the second half of 2020, 93% of the vaccinated nursery piglets examined at the end of the battery age are seronegative, but even in this group, we found 6 animals having shed the herd’s wild-type resident virus. Consequently, all pigs of this group were removed from the farm. By 2021, the results allowed the decision to terminate the ongoing vaccination of pigs at the end of the battery, when they are installed in the fattening barn. The first fattener pigs that did not receive a vaccine were in January 2021.

After the second half of 2021, we did not identify any pre-weaned and pre-fattening pigs with positive PCR test results (36 pre-weaned piglets and 33 nursery pigs PCR positive in 2021 were found in the first half of the year). Among fatteners, the rate of negative PCR test results was 98% in both 2021 and 2022. In the remaining cases, the infecting virus was the used vaccine in the herd. Serological tests gave positive results in 7–9% of samples. The reason behind the increased seropositivity was the failure of staff members who implemented the immunization did not comply with the internal epidemic prevention instructions (including change of clothes). This resulted in the spread of the vaccine virus and the accompanying sero-conversion in a single fattening room. As a result of ongoing control measures and based on the available test results the regional authorities classified the farm as a vaccinated herd free of PRRS in October 2021.

## 4. Discussion

Stabilization of the breeding stock is the preferred approach in PRRS elimination programs in large breeding herds whenever replacement of the stock is not an option of choice. Based on the categorization of Holtkamp and co-workers [17], the basis for achieving a stable herd is that the PRRS RT-PCR tests of 30 selected pigs sampled at 30-day intervals become negative for 4 months. A main feature of stable herds is that PRRS seropositive sows farrow PRRSV-negative piglets [17,18,26]. The most used method is that breeding animals on the farm are immunized with a live, attenuated vaccine virus or infected with a live herd-specific virus strain [27,28]. Vaccination or artificial infection is combined with strict internal and external biosecurity measures, and at the same time, the herd is kept isolated [29]. As far as we know, the method has only been used in a few cases in Europe. For example, Berton et al. (2017) conducted surveys on the stabilization of PRRS status on a farrow-to-finish type pig farm in France’s most intensive pig-rearing region [13]. The vaccination protocol consisted of two-dose vaccinations of the breeding herd with an interval of 1 month, followed by a repetition with an interval of 16–20 weeks. In the case of the weaned pigs, laboratory monitoring was carried out with a sample number of 30 pigs per batch. This method was successful in 15 out of 23 farms. The lack of success, in a small fraction of cases, was explained by violation of the epidemic prevention measures tracked back to the herd staff. The final conclusion of these authors was that it is possible to achieve stabilization of pig herds from farrow to slaughter regardless of their size and location.

In 12 American farrow-to-weaning type herds, Almeida et al. (2021) demonstrated that the distribution of PRRSV viraemic piglets in the farrowing pen is random among the litters, which is another significant challenge for the determination of the effective sampling method and the sample number [30]. Ultimately, decisions regarding the sample size and the selection of pigs to be sampled must be adapted taking into account the local circumstances. These authors modelled the sample size to detect at least one viraemic pig by fitting data to the prevalence of infection (with values of 25%, 20%, 15%, 10%, 5%, 4%, 3%, 2%, and 1%) at various confidence values (such as 90%, 95, and 99%). The models indicated that in order to find at least one PRRSV-infected piglet at the end of farrowing with 95% confidence, it is necessary to take 96 samples for 100 pigs, 130–154 for 200, 177–210 for 400, and 171–236 for 600 sows. These numbers are completely consistent with the prevalence table currently in circulation in our country.

In Hungary, where this study was carried out, an average farm differs from the farrow-to-wean type herd, and the most common type of breeding farm is farrow-to-finish. Consequently, PRRSV can spread not only within the same age groups but also between different groups in the phases of the fattening process, further complicating the progress of the elimination in our country. The National PRRS Eradication Committee, which coordinates the PRRS elimination program in Hungary, extended the elimination program with a new option when introduced the qualification of the PRRS-free, vaccinated herd in 2019. The criteria for creating such a herd are that the breeding herd is stable, the weaned piglets always show a negative result by PCR test, and the fattening herd is seronegative when slaughtered. In the case of weaned piglets, it requires a laboratory test control with 95% confidence and 2% prevalence. For example, on a 1000-sow farm, with 180 farrowing per month and with 12 piglets per farrowing, out of the 2200 piglets born, the sampling size of 3 pigs/farrowing well exceeds even the 95% confidence level and the 1% prevalence level. This practice is therefore fully supported by the latest studies by Almeida and co-workers, which are based on large-scale sampling [30].

In the case of the 1 sample/litter method, each sample was individually tested by PCR, for the 3 samples/litter method the samples were combined into a pool resulting in no cost difference. Only samples with positive PCR results were tested by the DIVA PRRS PCR test, therefore, no significant additional cost was incurred. Detection and culling of wild-type virus-positive sows and their offspring ensure steady production, while full replacement of an entire stock could interrupt the production for up to 1–1.5 years. However, the practice of depopulation and repopulation involves fewer risks and demands less attention and meticulous work.

## 5. Conclusions

In this study, we proved that the sampling method for declaring litters free of PRRSV is very important as we found significantly more PRRSV-positive litters when three weaned piglets/litter were examined. This means when a lower than required number of samples is tested, the status of the weaned piglet population could be misinterpreted. Applying this sampling approach and using laboratory methods that quickly detect the presence of the wild virulent virus in the herd during the rearing period (DIVA PCR), and culling the PCR-positive litters and their sows significantly decreased the presence of the resident virus. Finally, we implemented the above-mentioned procedures on a farrow-to-finish type farm and successfully prevented the infection of different age groups with the wild-type PRRSV. The results presented here indicate that applying these measures and strict biosecurity measures facilitates the PRRS-free status to be achieved in farrow-to-finish herds within two years.

## Figures and Tables

**Table 1:** PCR and ELISA results of Farm “A” using one piglet per litter and 3 piglets per litter for diagnostic investigations.

Period	Blood Samples from 4-Week-Old Piglets	PRRSV PCR Positive	Wild-Type Virus Positive	Vaccine Virus Positive	ELISA Positive (Piglets)
1/litter PCR	723 (241 litters)	0	0	0	56 (7.75%)
3/litter PCR, DIVA PCR	2508 (836 litters)	60 (20 litters) (2.39%)	3 (1 litter) (0.12%)	57 (19 litters) (2.27%)	87 (3.47%)

**Table 2:** PCR and ELISA results of Farm “B” using 3 piglets per litter for diagnostic investigations from different age groups.

Age Group	Number of Serum Samples	PRRSV PCR Positive	Wild-Type Virus Positive	Vaccine Virus Positive	ELISA Positive (Piglets)
Weaned piglets (3/litter)	3349 (1116 litters)	12 (4 litters) (0.36%)	3 (1 litter) (0.09%)	9 (3 litters) (0.27%)	2241 ° (79.86%)
35-day old	1438	17 (1.18)	1 (0.07%)	16 (1.11%)	848 (58.97%)
67-day-old	1510	79 (5.23%)	14 (0.93%)	65 (4.3%)	617 * (43.36%)

° From 3349 serum samples, 2806 were tested with ELISA. * From the 1510 serum samples, 1423 were tested with ELISA.

**Table 3:** PCR results of Farm “C” using 3 piglets per litter for diagnostic investigations before and after the culling of PRRSV-positive farrowed sows.

Period	Blood Samples from 3-Week-Old Piglets	PRRSV PCR Positive	Wild-Type Virus Positive	Vaccine Virus Positive
Before culling wild-type PRRSV positive farrowed sows	8910 (2970 litters)	96 (32 litters) (1.08%)	78 (26 litters) (0.88%)	18 (6 litters) (0.2%)
After culling wild-type PRRSV positive farrowed sows	9906 (3302 litters)	141 (47 litters) (1.42%)	0 (0%)	141 (47 litters) (1.42%)

**Table 4:** PRRS laboratory analyses of serum samples originated from Farm “D” from January 2020–August 2022.

Method		Preweaned Piglets (3 Piglets/Litter)			Nursery Pigs			Slaughtered Pigs		
		2020	2021	2022	2020	2021	2022	2020	2021	2022
ELISA										
	total	4761	5050	3456	1930	3058	1977	1632	5277	3721
	negative	2914 (61%)	2764 (55%)	2533 (73%)	1803 (93%)	3027 (99%)	1977 (100%)	515 (32%)	4902 (93%)	3377 (91%)
	positive	1847	2286	923	127	31	0	1117	375	344
PCR										
	total	4761	5050	3456	1930	3058	1977	1632	5277	3721
	negative	4389 (92%)	5014 (99%)	3456 (100%)	1639 (85%)	3025 (99%)	1977 (100%)	1395 (85%)	5157 (98%)	3654 (98%)
	positive	372	36	0	291	33	0	237	120	67
DIVA-PCR										
	total	372	36	0	291	33	0	237	120	67
	negative	9	0	0	6	6	0	0	0	0
	vaccine virus	357 (96%)	36 (100%)	0	279 (96%)	27 (82%)	0	237 (100%)	120 (100%)	67 (100%)
	resident virus	6 (1.6%)	0	0	6 (2.1%)	0	0	0	0	0

## Data Availability

Detailed data are available from István Szabó via the e-mail address shown on the title page.
